# Modelling clinical experience data as an evidence for patient-oriented decision support

**DOI:** 10.1186/s12911-020-1121-4

**Published:** 2020-07-09

**Authors:** Junyi Yang, Liang Xiao, Kangning Li

**Affiliations:** 1 School of Computer Science, Hubei University of Technology, Wuhan, Hubei China; 2 Wollongong Joint Institute, Central China Normal University, Wuhan, Hubei China

**Keywords:** Clinical evidence, Clinical decision support, Patient experience, Social networks, Sentiment analysis

## Abstract

**Background:**

Evidence-based Clinical Decision Support Systems (CDSSs) usually obtain clinical evidences from randomized controlled trials based on coarse-grained groups. Individuals who are beyond the scope of the original trials cannot be accurately and objectively supported. Also, patients’ opinions and preferences towards the health care delivered to them have rarely been considered. In this regards, we propose to use clinical experience data as an evidence to support patient-oriented decision-making.

**Methods:**

The experience data of similar patients from social networks as subjective evidence and the argumentation rules derived from clinical guidelines as objective evidence are combined to support decision making together. They are integrated into a comprehensive decision support architecture. The patient reviews are crawled from social networks and sentimentally analyzed to become structured data which are mapped to the Clinical Sentiment Ontology (CSO). This is used to build a Patient Experience Knowledge Base (PEKB) that can complement the original clinical guidelines. An Experience Inference Engine (EIE) is developed to match similar experience cases from both patient preference features and patient conditions and ultimately, comprehensive clinical recommendations are generated.

**Results:**

A prototype system is designed and implemented to show the feasibility of the decision support architecture. The system allows patients and domain experts to easily explore various choices and trade-offs via modifying attribute values to select the most appropriate decisions.

**Conclusions:**

The integrated decision support architecture built is generic to solving a wide range of clinical problems. This will lead to better-informed clinical decisions and ultimately improved patient care.

## Background

Evidence-based Clinical Decision Support Systems (CDSSs) are usually developed on the basis of the best clinical available evidence and could effectively interpret clinical data at the point of care. This may assist clinicians in keeping their knowledge up-to-date and delivering improved care [1]. In particular, studies of the “new generation” CDSSs demonstrate that they have the potential of addressing problems in clinical practice, decreasing the rate of medication errors, and increasing patients’ adherence to healthcare. The CDSSs are promising in promoting evidence-based medicine, yet there is a lot of work to be done to achieve the expected benefits [2].

Most of the literatures at the moment focus on improving the clinical outcome of ordinary people through the design and development of CDSSs. It was rarely recognized that evidence-based CDSSs limit their applicability to particular patient conditions. In fact, evidence-based clinical knowledge such as clinical guidelines can only cover about 80% of clinical situations, and the remaining cases must be dealt with case by case [3]. Moreover, patient values and preferences need to be respected in considering treatment options and other interventions [4, 5]. Public health reports [6] re-emphasized the importance of patient-centric care and suggested the incorporation of patient preferences into decision-making processes. Therefore, this work is motivated by the challenges as follows.

1. The evidence-based medicine gains its evidence from randomized controlled trials, which are based on coarse-grained groups. Hence, it is impossible to provide an accurate and objective assessment upon an individual who falls outside the scope of the original trials.
2. Even the evidence of recommending a clinical decision for a patient under consideration is well included, the patient may have her own particular preferences that differ from all the others. It is usually the case that such variations are not sufficiently communicated between clinicians and patients to deliver customized and joint decisions.

United States Preventive Services Task Force (USPSTF) and the American Cancer Society (ACS) recommend that patients and physicians participate in shared decision making [7]. As primary stakeholders of clinical decisions, patients have not yet been given adequate consideration in the CDSS system development. Fortunately, researchers have increasingly realized the importance of patient-centered CDSSs in achieving high quality healthcare. James G. Dolan proposed a multi-criteria decision analysis model [8] that incorporates multiple considerations into the decision-making process to help patients better understand and insight into the decisions they are confronted. Lucia Sacchi et al. presented the theoretical, technological and architectural aspects of a framework [9] that encapsulates decision models and instruments to elicit patients’ preferences into a single tool, thus enabling physicians to exploit evidence-based medicine and shared decision-making in the same encounter. Other approaches [10–14] take into account various factors, e.g. safety, quality and consistency of evidence, and affordability of the overall treatment in a patient-centered perspective. Nevertheless, an important issue yet remains to be solved even given the consideration of all these factors: whether a decision based on the best clinical evidence available is the most appropriate decision to a particular patient? Current research demonstrates that different preferences and values influence decisions differently [15]. For example, a musician may deem the loss of the ability to play an instrument due to neuropathy worse than the loss of survival but may be less concerned about other side effects. Similarly, one elderly patient may place a high priority on extending survival at the expense of toxicities and quality of life to attend his granddaughter’s pending wedding. The specific relationship between preferences and decision-makings depends on patients’ cognitive biases [16]. Such decisions are ideally suited to being facilitated via decision support tools, but interventions that incorporate preferences either preceding or following the use of decision aids are very sparse.

One way that patient preferences may be incorporated into CDSSs is the consideration of similar patient experience data from healthcare social media. Studies show that patients use social media frequently to understand how other patients have been treated and what outcome they have achieved. It is reported in [17] that four out of five users are using the Internet to find personalized healthcare information related to particular diseases and their treatments. By knowing more about the clinical experience of other patients, people will be more prepared to their own treatments. Patient experience from social media includes patient opinions towards any clinical procedure they have gone through. The characteristics of each patient, such as individual needs, preferences, and emotional status, are relevant in decision-making. Current research suggests the mining of valuable data from social networks can be useful. Halder et al. proposed a system [18] that monitors health conditions, emotions and interests of patients from patients’ tweets and emails. Adetola et al. proposed a new framework [19] for linking social media, intelligent agents and expert systems to support the formulation of open innovation strategies. Unfortunately, such valuable works have not been integrated into the evidence-based medicine to deliver the best possible care suited to patients.

In this paper, we propose the modelling of clinical experience data as an evidence for patient-oriented decision support. A comprehensive decision architecture is constructed to introduce subjective experience data of patients upon the existing the rule-based objective decision support architecture. The major architecture is addressed in the methods section and the method of integrating the necessary components is described. In the results and discussions sections, a prototype system is designed and implemented, and the feasibility of the decision support architecture is verified by the case study of triple evaluation of breast cancer.

## Methods

### An overview of the architecture and its major components

**Fig. 1:**
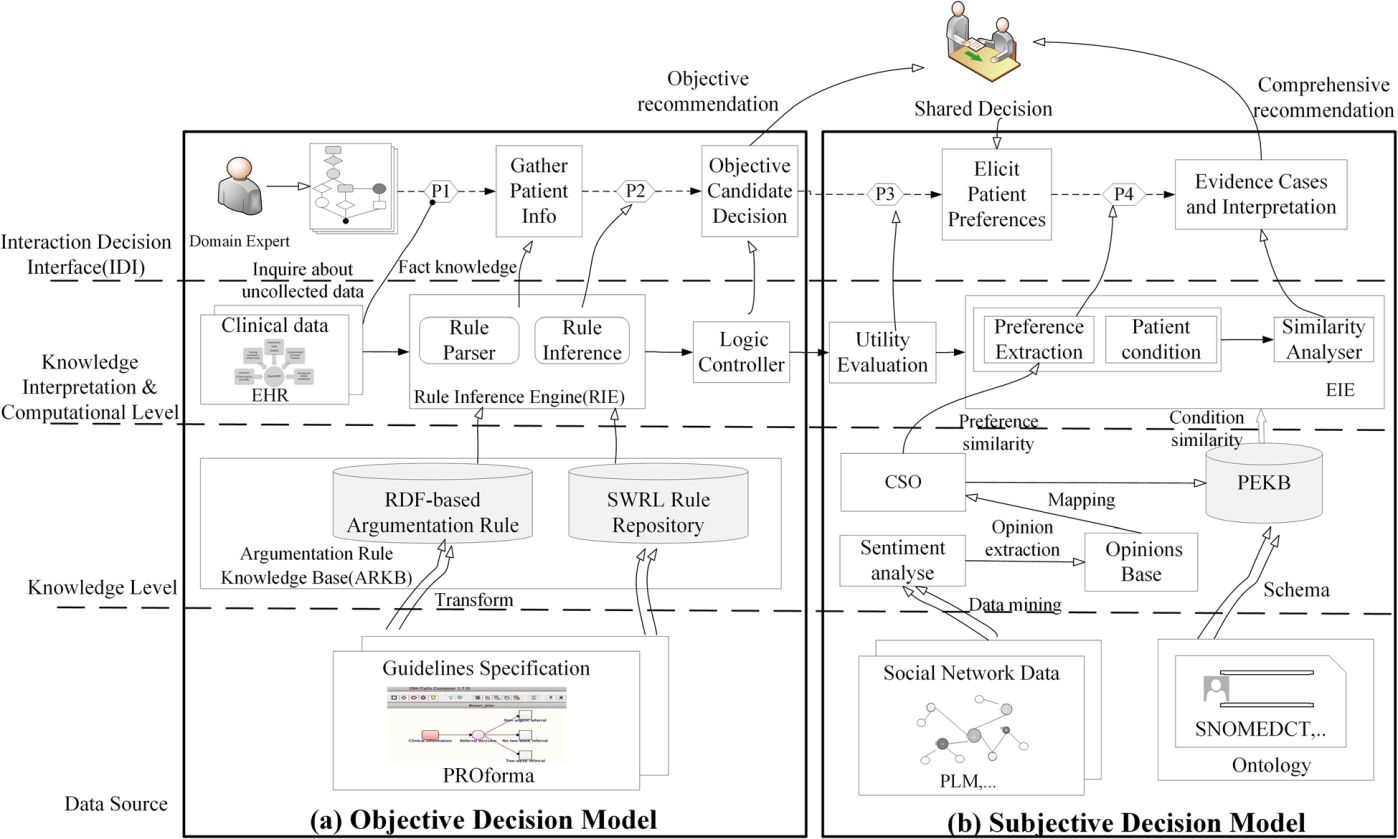
A clinical decision support architecture for modeling patient experience as evidence

A comprehensive decision architecture is proposed here, which takes into account the subjective preferences of patients based on objective clinical decision support to provide a more comprehensive recommendation. A conceptual architecture is shown in Fig. 1. The original distributed decision support part of the shown in the part (a) of the Fig. 1 and can be referenced in our previous work [20, 21]. First, clinical guidelines must be explicitly modeled and should be considered as a set of argumentation rules in that part of the architecture. Second, argumentation rules are represented in the form of Resource Description Framework (RDF) [22] triples, which helps semantic reasoning and automated updating of clinical knowledge. Finally, the clinical scenarios are matched where argumentation rule are applied to the Electronic Health Record (EHR). A set of predefined Semantic Web Rule Language (SWRL) [23] rules are reasoned by Drools [24] and recommendation are generated automatically at the point of care. The core components and their interactions in the architecture after incorporating the patient experience are shown in part (b) of Fig. 1. First, patient reviews are crawled from the scattered social network, which are processed using a sentiment analysis tool to generate a set of structured data. Then, the results are mapped into the Clinical Sentiment Ontology (CSO) to build the Patient Experience Knowledge Base (PEKB). An Experience Inference Engine (EIE) is designed to match the patient preference characteristics to the experience cases that meet current patient’s condition via calculating the similarity, and weight the candidate objective decision to obtain a set of the reordered comprehensive decisions. The best a set of experience cases are recommended through the EIE that reasons and calculates similarity from conditions and preferences of patient based on PEKB. An Interactive Decision Interface (IDI) is designed to elicit patient preferences and allows clinical experts and patients to together choose the most optimum decision with a well understanding of decision recommendations---the trade-off of various choices via exploring preference similarities. Finally, new patient experience cases are collected as the learned experience which is stored in the PEKB to achieve self-learning.

### Patient experience Knowledge Base (PEKB)

#### Clinical sentiment ontology (CSO)

The concepts of a CSO that is used to describe the patient’s opinions or experiences can be grouped in three main categories:

**Table:** 

**Fig. 2:**
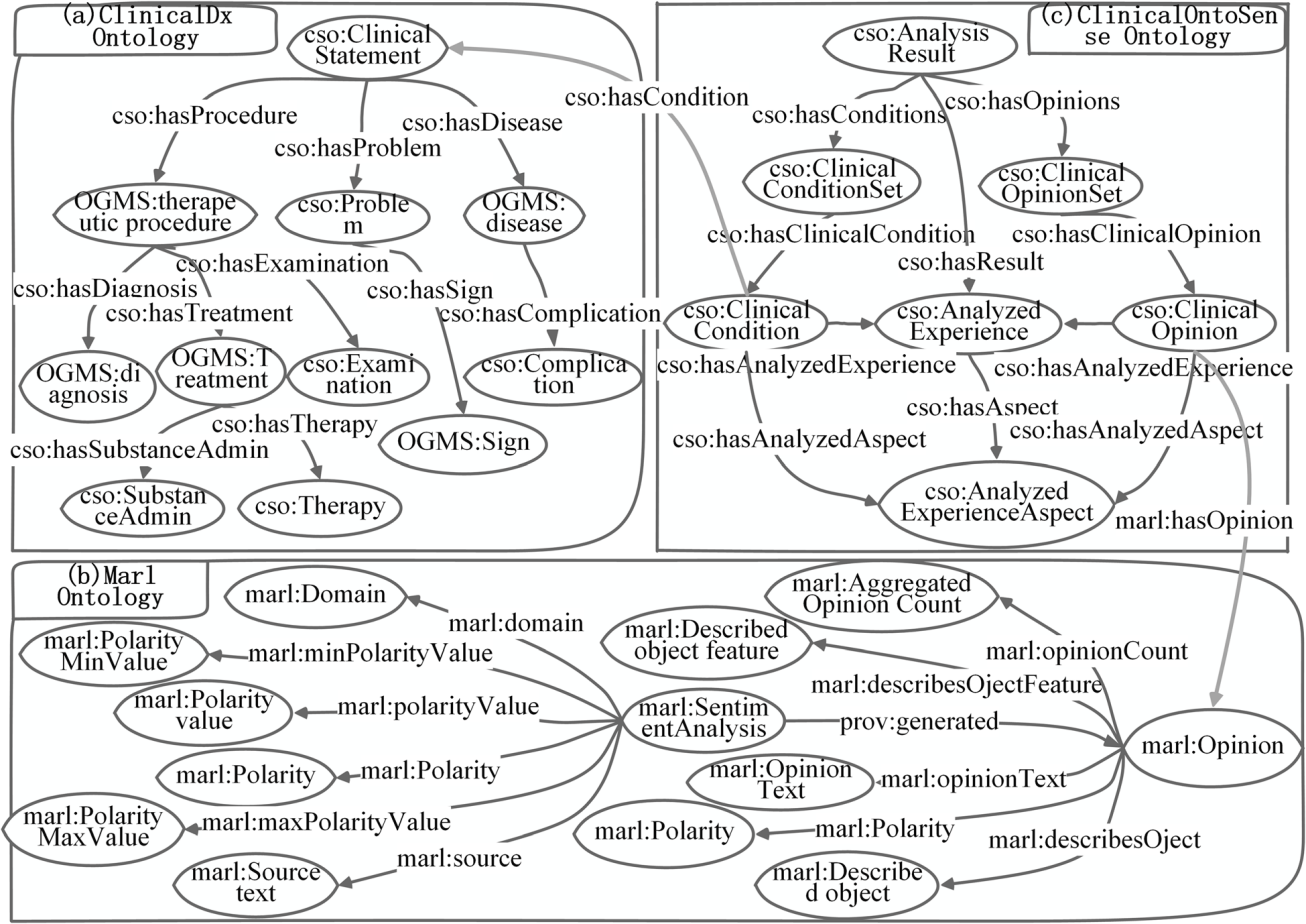
Clinical Sentiment Ontology (CSO)

• concepts that express human sentiment and the tendency to opinions; • concepts that describe specific clinical knowledge; • concepts that provide a connection between the expression of sentiment and the tendency to opinions, and analyzed clinical concepts and its properties;	

For the first category of concepts, the ones describing sentiment are found in the scientific literature, and for the last two categories, no appropriate ontology was identified. Therefore, a specific clinical diagnosis ontology modelling the relations between clinical feature, diagnosis and their associated properties had to be created. Afterwards, a clinical sentiment ontology, named ClinicalOntoSense, which connects the expressed sentiments, the tendency to opinions and the analyzed experienced and their aspects was defined. The main concepts from the three ontologies and the object properties connect them are shown in Fig. 2.

A new clinical diagnosis ontology is proposed for which the main classes and properties are shown in part (a) of Fig. 2. The *cso* prefix is used in the paper to denote classes or properties belonging to CSO ontology. As shown in [25], several generic widely used vocabularies for annotating the data extracted from medical science currently exist. One of is the Ontology for General Medical Science (OGMS) [26], used to ontological treatment of disease and diagnosis and on carcinomas and other pathological entities. The ClinicalDX proposed reuses several properties including *OGMS:therapeutic* procedure, *OGMS: disease* and *OGMS:diagnosis*. Moreover, the classes such as *cso: Problem* and *cso: Complication* and *cso: Therapy* are added additionally, which are defined in the ClinicalDx ontology.

Marl [27, 28] is proposed as an ontology-based representation of sentiment analysis, and annotates and describes the subjective opinions expressed on the network. *Marl: SentimentAnalysis* is a class that analyses a source text (*marl: Sourcetext*) according to an algorithm and produces an *marl: Opinion* about the entities described (*marl: DesicribedObject*) in the source text. *Marl: Opinion* is a class that represents the results of a sentiment analysis process. The main features (*marl: DescribedObjectFeature*) of the extracted Opinion are the Polarity (positive, neutral or negative). The *marl* prefix is used in the paper to denote classes or properties belonging to this ontology, as shown in the part (b) of Fig. 2.

The application specific ontology, shown in part (c) of Fig. 2, describes the analyzed entities, like clinical conditions, clinical opinions, together with their aspects and the generated experience. The *cso: AnalysisResult* class provides the necessary link with the ClinicalDx ontology and Marl ontology, previously described. It includes the analyzed opinions tendency towards clinical experience, represented by *cso: AnalyzedExperience* and its aspects, represented by *cso: AnalyzedExperienceAspect*. The main classes, around which the ontology is built are *cso: ClinicalConditionSet* and *cso: ClinicalOpinionSet*. The *cso: AnalysisResult* class serves as a base class for *cso: ClinicalConditionSet* and *cso: ClinicalOpinionSet* and defines the *cso:hasConditions* and *cso:hasOpinionSet* object property, containing the keywords or hashtags that will be used to retrieve the analyzed experience. The analyzed entity is modeled by the *cso: AnalyzedExperienc*e class, representing the subjective opinions for a specific clinical condition. Given the fact that people usually express opinions not only about the concept, but also about its characteristics the *cso: AnalizedExperienceAspect* class models the relevant characteristics.

#### Sentiment Analysis & Opinion Mining

We use the sentiment analysis module built in NLTK to determine positive, negative and neutral emotions in sentences from the preprocessed dataset. The results of sentimental analysis are used as the source data of opinion mining.

An opinion consists of two key components [29]: a target and a sentiment on the target, where the target can be any entity and the sentiment is a numeric rating score expressing the intensity of the sentiment. First, all the nouns and noun phrases are found in the tagged dataset, and association mining [30] is used to find frequent feature [31] sets. Then, the adjectives closest to the frequent features are extracted as opinion words in each sentence. 30 adjectives with sentiment polarity are pre-stored in a seed list, as a set of opinion words of known polarity. For each opinion word, synonyms of the opinion word are searched in WordNet [32]. If the synonyms exist in the seed list, the sentiment polarity of the synonyms can be found, and the opinion word is set to the same sentiment and added to the seed list. If the synonyms do not exist in the seed list, the antonyms will be searched. If the antonyms are in the seed list, the opinion word is set to the opposite sentiment and added to the seed list. An opinion word that its synonyms or antonyms cannot be found, is considered to be an invalid word. The process is iterated until the size of the seed list is no longer expanded. The opinion or sentiment of each frequent feature can be calculated for each sentence in the dataset.

**Fig. 3:**
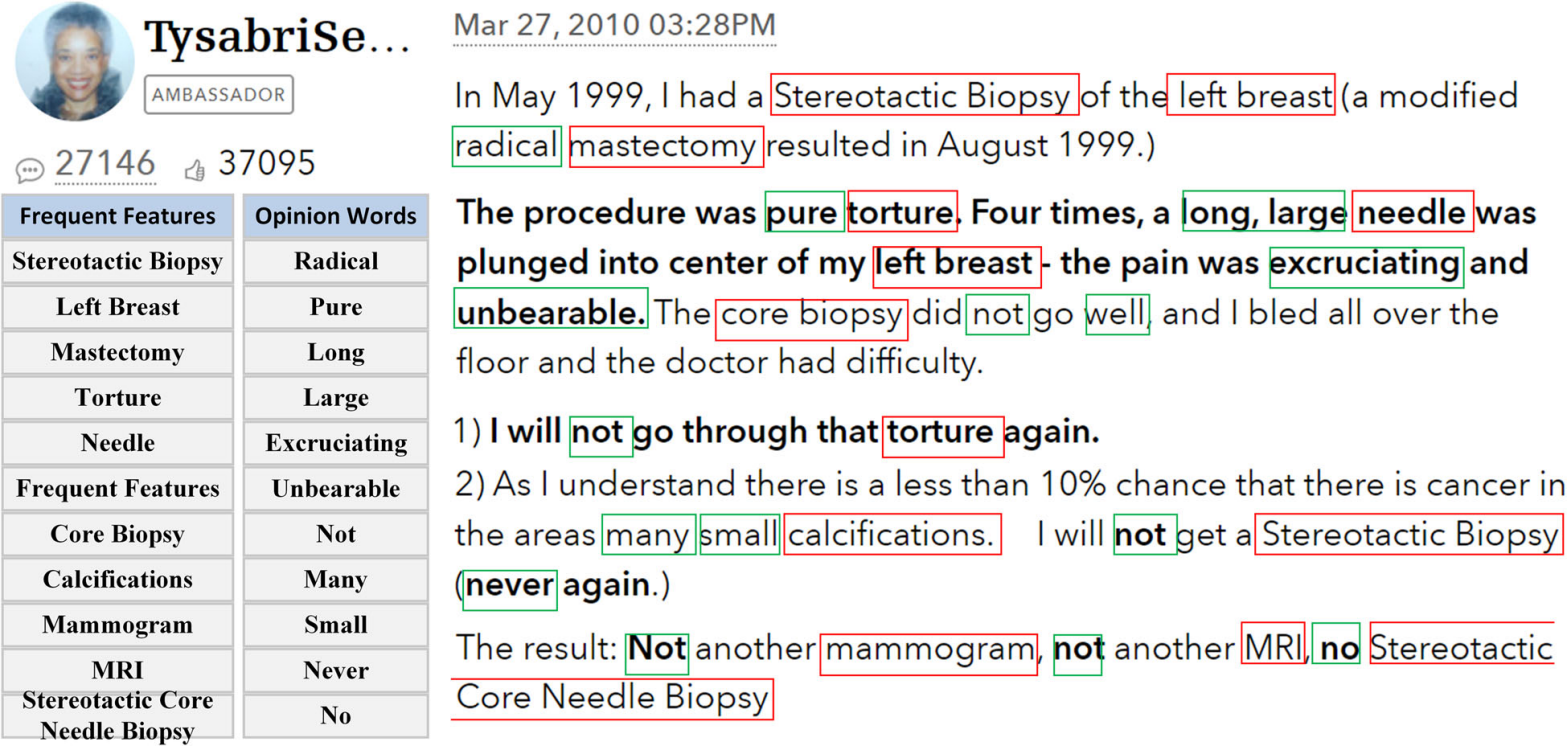
Frequent features and opinion words from PLM

Patients Like Me (PLM) [33], a social network, is used to collect sentiment comments about health care. Through the website, patients connect with others who have the same disease or condition and track and share their own experiences with the goal to improve outcomes. The frequent features and the opinion words analyzed in a specific example from a patient’s opinion in the PLM is shown in Fig. 3. The frequent features calculated from the tagged nouns and noun phrases are “Stereotactic Biopsy”, “Left Breast” and “Mastectomy” etc. Subsequently, the associated opinion words can be found through WordNet matching such as “Radical”, “excruciating” and “Unbearable” etc. Sentiment polarity can be calculated when frequent features and associated opinion words are combined as opinions.

**Fig. 4:**
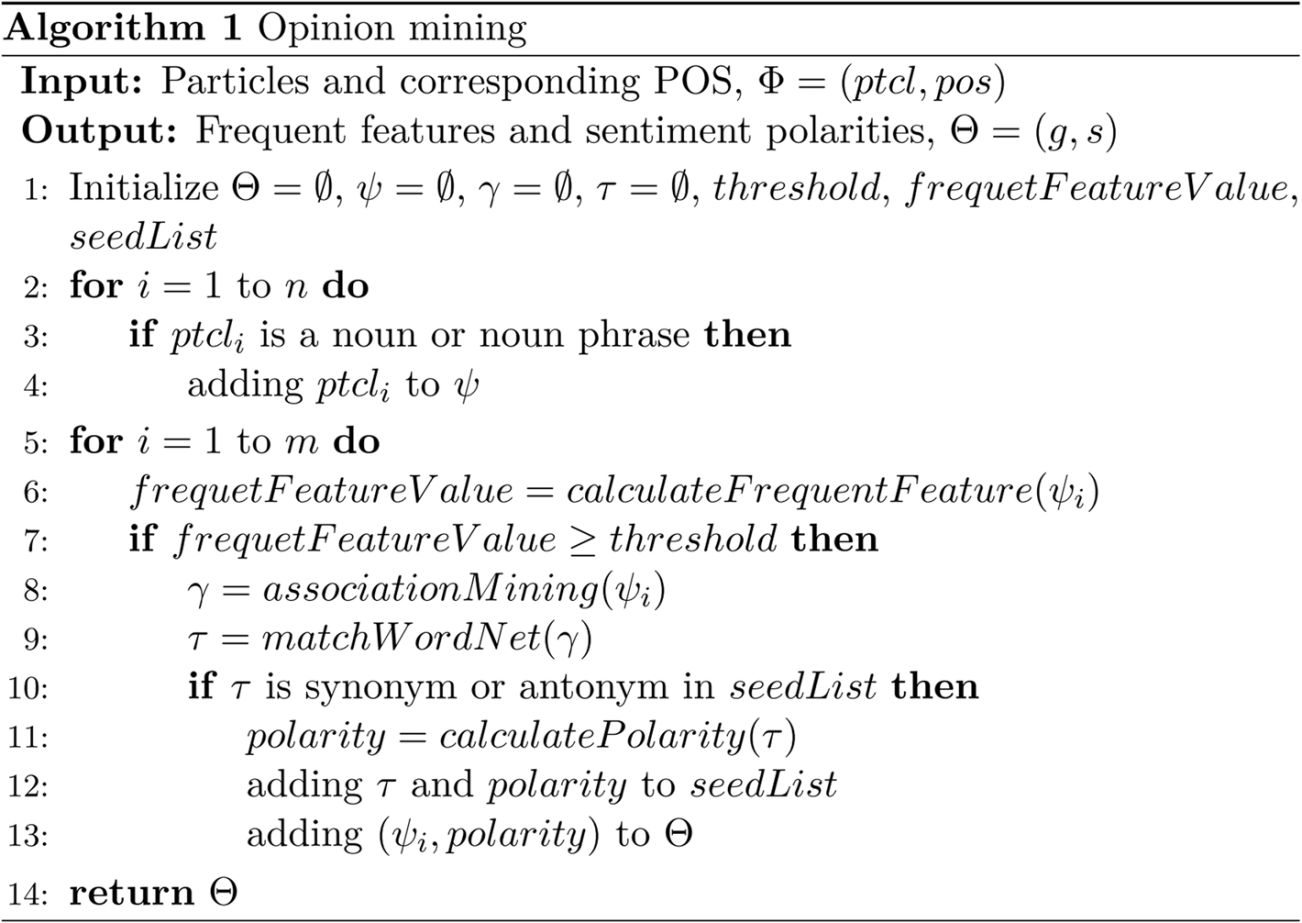
The pseudo code of opinion mining algorithm

The pseudo code of the opinion mining algorithm is shown Fig. 4, the input is a set of particles and their POS, *Φ*. The output is a set of opinions for frequent features, denoted as *Θ*. The nouns and noun phrases of the dataset are represented as *ψ*. The synonyms and antonyms found from WordNet are stored in *τ*. Moreover, *threshold* is the pre-defined minimum support value which is used to judge whether a noun or noun phrase can be a frequent feature. The frequent feature value of each element in *ψ* can be calculated as *frequentFeatureValue*. Pre-defined adjectives of obvious sentiment (polarity) are set in a seed list, as a set of opinion words of known polarity, denoted as *seedList*.

The mapping from the result of opinion mining to ontology which helps semantic reasoning and automation of CDSSs is shown in Fig. 2. First, the domain of opinion matches clinical disease which is the basis of the opinions mined from the dataset. The opinions of patients can reflect the features and severity of the symptoms. Besides, patients often comment on some therapies and examinations they have experienced in PLM. The opinions mined from the comments can also show the effects of the therapies and examinations to some extent.

**Fig. 5:**
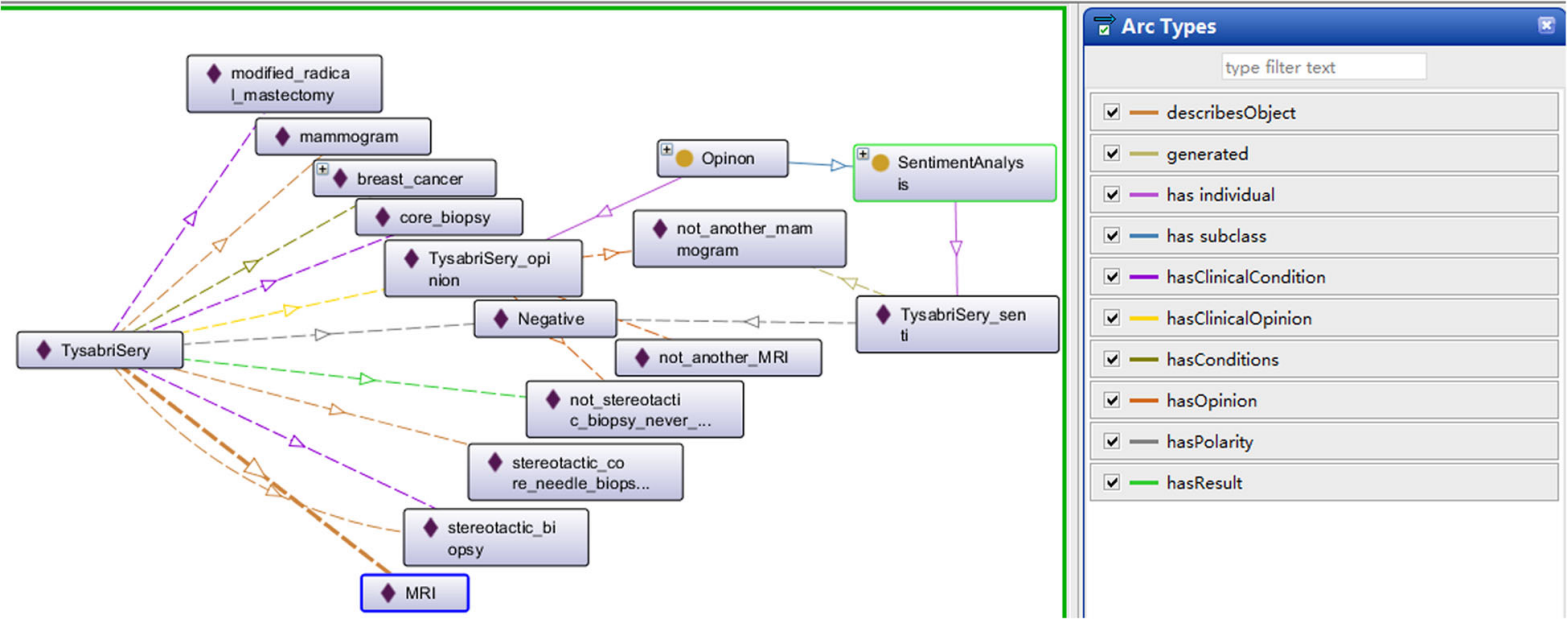
An example of sentiment analysis based on CSO

The screenshot of some of the ontology individuals shown in Fig. 5 corresponds to the specific example shown in Fig. 3. The clinical condition (*cso:hasCondition*) of the patients was confirmed as breast cancer and modified radical mastectomy was carried out. The current clinical procedure (*cso:hasProcedure*) is stereotactic biopsy of the left breast. The patient’s sentiment polarity (*marl:hasPolarity*) for the description object (*marl:describesObject*) is negative, and the more subjective feeling is “I will not go through that torture again”. The resulting opinion (*marl:hasOpinion*) is that “the pain was excruciating and unbearable, stereotactic biopsy is not recommended”.

### Experience inference engine (EIE)

**Fig. 6:**
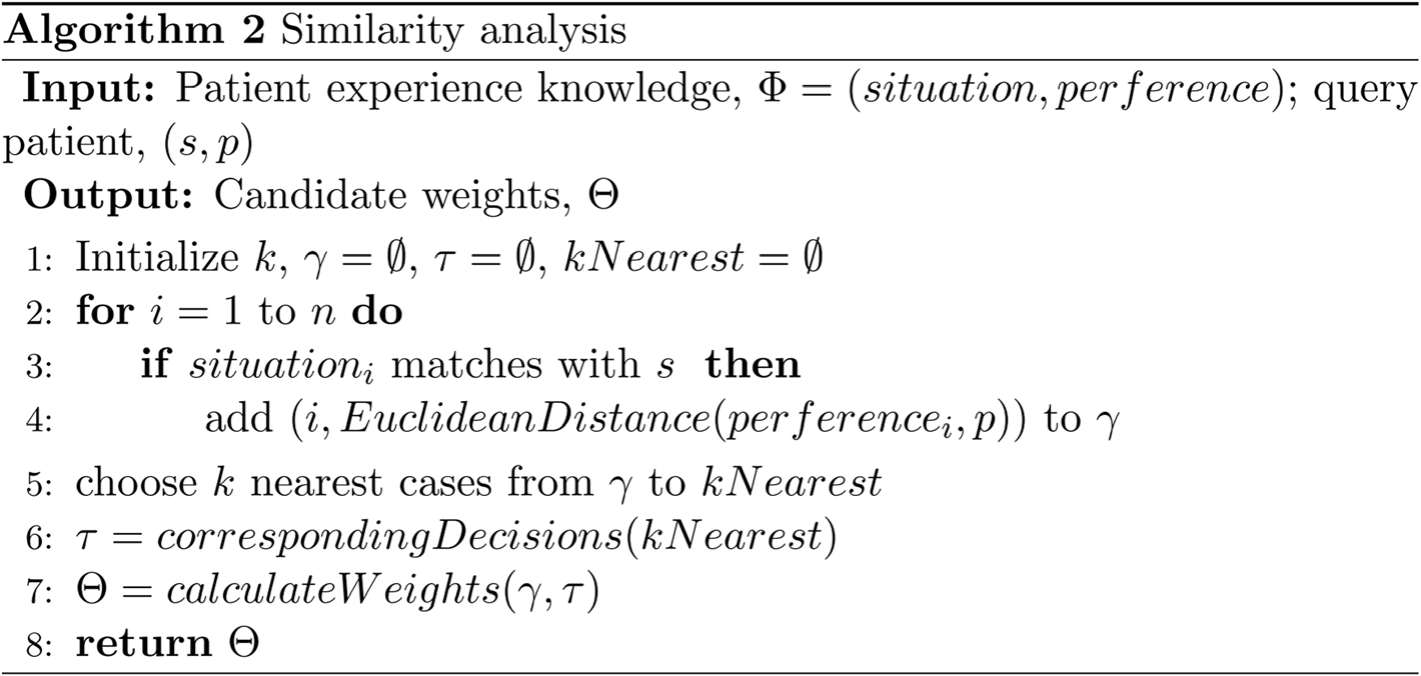
The pseudo code of similarity analysis algorithm

The features of patients can be divided into two aspects which are situation of patients and preference of patients. Patient similarity analysis is based on “local” and “global” similarity retrieval algorithms included in KNN (k-NearestNeighbor) [34]. The local similarity means to measure distance of the situation or preference of patients between the query case and the patient experience knowledge. The global similarity is similarity of all attributes consisting of situation and preference of patients. The algorithm is shown in Fig. 6.

The input of Algorithm 2 is the experience knowledge from PEKB and the conditions and preferences of current patient, and the output is the probability of similarity between the two. K is a hyper-parameter of KNN set by experience to decide the number of cases being picked. First, the condition of the current patient needs to be matched with the condition of cases in PEKB. Then, the Euclidean distances are calculated on patient preference between the condition matched cases and the current patient. The k nearest cases are selected from all cases. Finally, the weights of the k candidates are calculated.

## Results

### System Design & Implementation

To implement our designed idea which is to model clinical experience data as an evidence for patient-oriented decision support, we developed a CDSS with patient experience as an evidence support which is validated on a case of breast cancer by triple assessment to make better-informed decisions and improve care and patient compliance in various conditions.

**Fig. 7:**
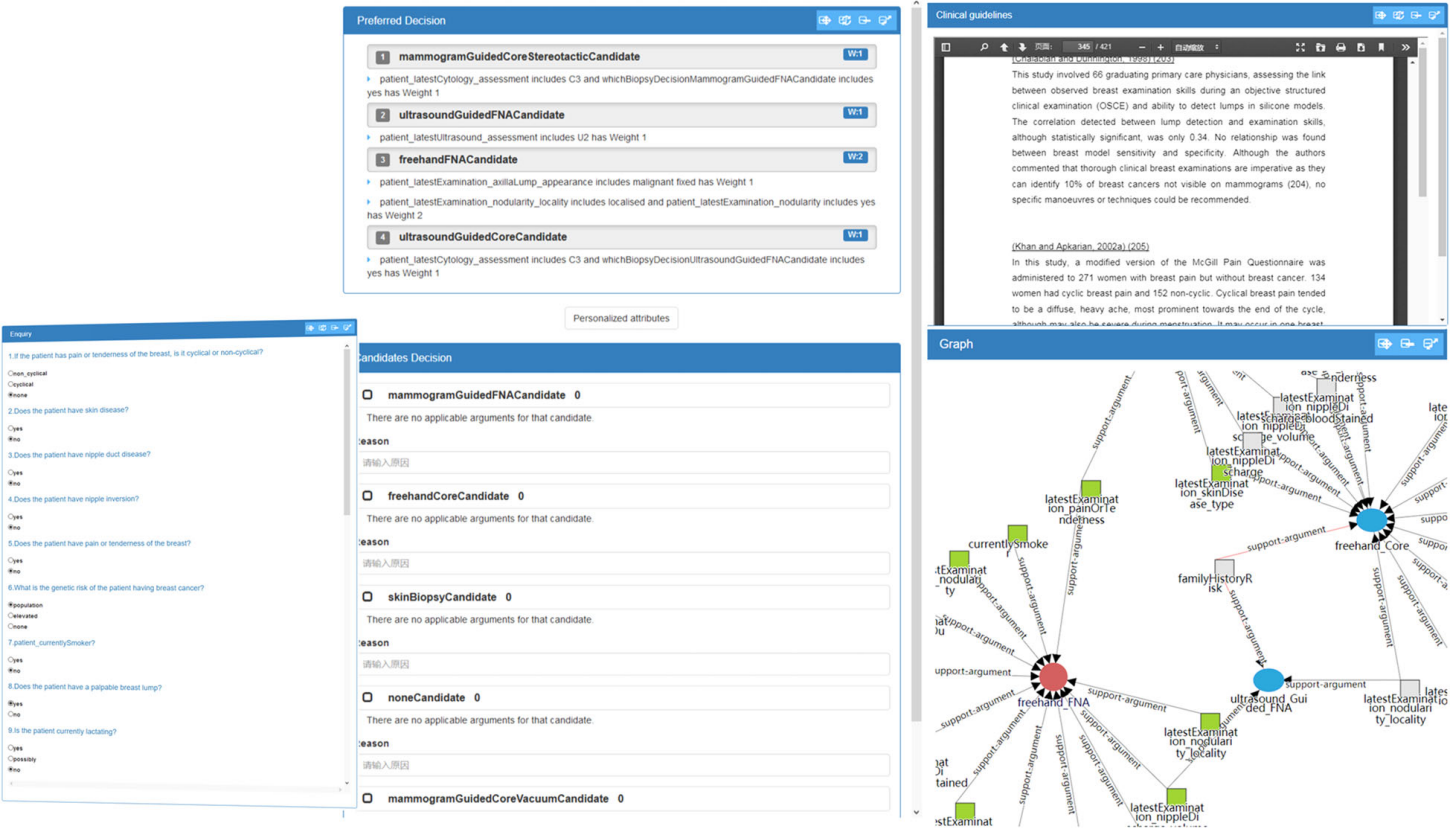
Objective Decision Interaction Interface

The objective IDI of the implemented CDSS is shown in Fig. 7 including five parts which are enquiry, clinical guidelines, preferred decision, objective candidate decision and knowledge graph. First, patients need to answer the listed questions in the enquiry part, which is a necessary step for data collection in the CDSS. Patients can also look up to the clinical guidelines which can be automatically turned to corresponding section when they click the link of the questions for clinical knowledge explanation. The questions are mapped to the nodes of the knowledge graph generating candidate decisions after the questions are answered, and the corresponding nodes are also lighted. Knowledge graph can also help domain experts to understand and enhance their knowledge of clinical arguments to make better decisions. The gathered patient information (answers of the questions) needs to be parsed and inferred in the rule inference engine to generate reordered objective candidate decisions and preferred decisions. The nodes presenting preferred decisions in knowledge graph are red, and standing for evidences supporting preferred decisions are green.

**Fig. 8:**
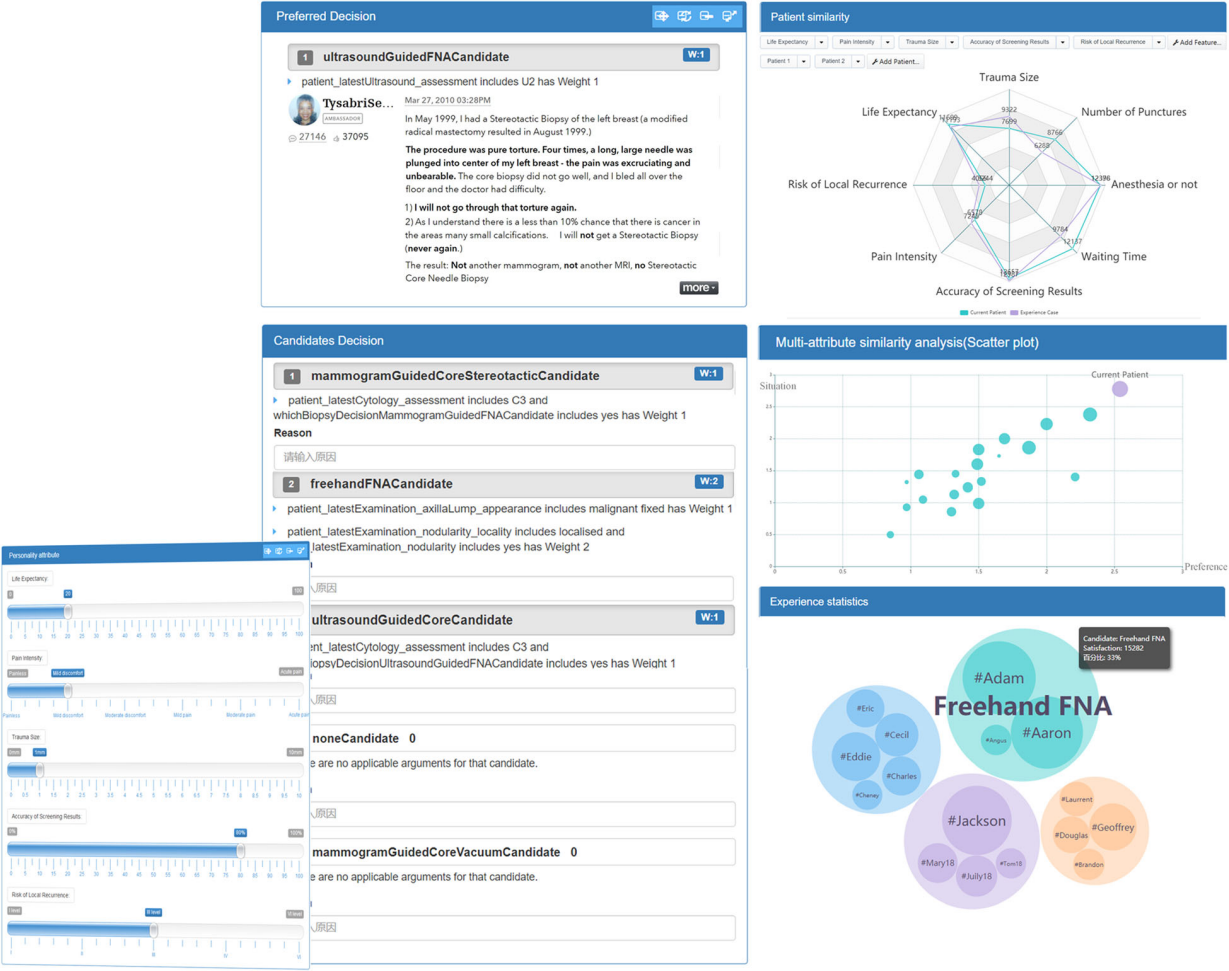
Subjective Decision Interaction Interface

On the other hand, patient subjective evidence is also considered in the CDSS, which is shown in Fig. 8. There are six parts consisting of subjective IDI which are personality attribute, preferred decision, candidate decision, patient similarity, multi-attribute similarity analysis and experience statistics. Patient preferences can be drawn forth by the quantitative representation of the visual analog scale (VAS) [35] in the personality attribute part. There are several anchors of the VAS which are used to measure the extent of each patient attribute. Patients are required to rank each attribute in order of priority based on their personal preference for the attributes. Then, the preferred decision can be made according to patient preferences and situations including objective and subjective evidences. The candidate decisions can also be made to help patients to make more suitable decisions. In patient similarity part, the most similar experience case is calculated in PEKB, and patients can modify their preference attributes to dynamically re-calculate the most similar case. The experience case is compared with the current patient in terms of each patient preference attribute so that the patient can clearly see the differences between them. The current patient is not only compared with the most similar experience case, but the candidate cases according to patient preference and situation in multi-attribute similarity analysis part. For experience statistics, the nodes containing small nodes represent the objective preferred decisions shown in Fig. 7. The small nodes in the nodes of objective preferred decisions are patients who have made the same decisions, and the current patient can hover on the small nodes to view details of the patient.

## Discussion

In this paper, we discussed the importance of representing patient experience data as a significant contribution for evidence-based healthcare studies in CDSS. The outcomes obtained from our system reflect the impact of patient preferences on patient compliance and satisfaction. Our current research implies the importance of knowledge engineering of patient experience data to support futuristic patterns of clinical decision, which can be further extended to other specific healthcare studies as part of future work. In our work, we consider the PEKB ontology framework for disease conditions and patient sentiment, and PEKB can be enhanced to include other standard ontologies such as SNOMEDCT etc. The quality of the reference subjective experience ontology can influence the similarity of the expected outcomes. The dataset used for our evaluation was obtained from PLM for only one disease condition of breast cancer to demonstrate the methodology as a sample case study. PLM, in terms of its entirety, can be considered to provide disease progression trends for over 2700 conditions listed in its database. Likewise, we will consider to include practice-based and literature-based evidence with social network data to obtain an enriched inference.

## Conclusions

Overall, our current work aims to support patient experience as evidence in the CDSS, in conjunction with patient subjective preference to support patient-centered clinical decision making. Based on universal objective decision-making, patient subjective preference is introduced to solve the problem that the group test results cannot be adapted to the individual. We propose a novel method for constructing a knowledge base that constructs ontology and establishes relationships from both disease and emotions, and can assist in evidence-based decision making for diagnosis and treatment. Inference engine further supports reasoning among decision options and provides a mechanism for recommending a preferred option, being an appropriate diagnostic test, a treatment option, or a particular care pathway.

## Data Availability

The datasets used and analyzed during the current study are available from the first author upon reasonable requests.

## Abbreviations

CDSSs: Clinical Decision Support Systems
CSO: Clinical Sentiment Ontology
PEKB: Patient Experience Knowledge Base
EIE: Experience Inference Engine
USPSTF: United States Preventive Services Task Force
ACS: American Cancer Society
RDF: Resource Description Framework
EHR: Electronic Health Record
IDI: Interaction Decision Interface
SWRL: Semantic Web Rule Language
OGMS: Ontology for General Medical Science
NLTK: Natural Language Toolkit
PLM: Patients Like Me
POS: Part-Of-Speech
KNN: k-Nearest Neighbor
VAS: visual analog scale
